# Variation in inbreeding depression within and among *Caenorhabditis* species

**DOI:** 10.1093/g3journal/jkaf200

**Published:** 2025-08-29

**Authors:** Matthew V Rockman, Max R Bernstein, Derin Çağlar, M Victoria Cattani, Audrey S Chang, Taniya Kaur, Luke M Noble, Annalise B Paaby

**Affiliations:** Department of Biology and Center for Genomics & Systems Biology, New York University, New York, NY 10012, United States; Department of Biology and Center for Genomics & Systems Biology, New York University, New York, NY 10012, United States; Woven Health Collective, New York, NY 10038, United States; Department of Biology and Center for Genomics & Systems Biology, New York University, New York, NY 10012, United States; Department of Biology and Center for Genomics & Systems Biology, New York University, New York, NY 10012, United States; National Institute on Aging, Rockville, MD 20892, United States; Department of Biology and Center for Genomics & Systems Biology, New York University, New York, NY 10012, United States; Science Gallery International, Dublin 2, Ireland; Department of Biology and Center for Genomics & Systems Biology, New York University, New York, NY 10012, United States; Merck, Pune 411014, Maharashtra, India; Department of Biology and Center for Genomics & Systems Biology, New York University, New York, NY 10012, United States; EnviroDNA, Melbourne, Victoria 3056, Australia; Department of Biology and Center for Genomics & Systems Biology, New York University, New York, NY 10012, United States; School of Biological Sciences, Georgia Institute of Technology, Atlanta, GA 30332, United States

**Keywords:** inbreeding depression, *Caenorhabditis*, selfing, recessive genetic variation, mating-system transitions

## Abstract

Outbreeding populations harbor large numbers of recessive deleterious alleles that reduce the fitness of inbred individuals, and this inbreeding depression potentially shapes the evolution of mating systems, acting as a counterweight to the inherent selective advantage of self-fertilization. The population biological factors that influence inbreeding depression are numerous and often difficult to disentangle. We investigated the utility of obligately outcrossing *Caenorhabditis* nematodes as models for inbreeding depression. By systematically inbreeding lines from 10 populations and tracking line extinction, we found that inbreeding depression is universal but highly variable among species and populations. Inbreeding depression was detected across the life cycle, from mating to embryo production to embryonic viability and larval growth, and reciprocal crosses implicated female-biased effects. In most cases, the surviving inbred lines have dramatically reduced fitness, but the variance among inbred lines is substantial and compatible with the idea that inbreeding depression need not be an obstacle to the evolution of selfing in these worms. Populations of some species, including *Caenorhabditis becei,* exhibited modest inbreeding depression and could be tractable laboratory models for obligately outcrossing *Caenorhabditis*.

## Teaser

A lot of genetic variation is harmful and populations harbor different amounts of it. Recessive variation is a particularly important part of this story, as it can persist invisibly in outbreeding populations. Rockman et al. show that outbreeding relatives of *C. elegans* have lots of recessive deleterious variation, but species differ in the amount, and so do isolates within species. Highly inbred lines derived from these different sources vary in their fitness, and in some cases the fitness is high enough that an evolutionary transition from outcrossing to selfing would not be impeded by inbreeding depression.

## Introduction

The constant and unavoidable generation of damaging mutations saddles populations with a genetic load. This load consists of an expressed component, manifest as the reduction of fitness of an individual drawn from the population relative to that of a mutation-free individual, and a concealed component, due to recessive mutations, that manifests as inbreeding depression, a reduction of fitness in inbred relative to outbred individuals (Morton et al. 1956; Lynch and Walsh 1998; Charlesworth and Willis 2009; Hedrick and Garcia-Dorado 2016; Dussex et al. 2023).

Many aspects of population biology influence the distribution of expressed and concealed load. In small populations, deleterious alleles drift to high frequencies, resulting in higher expressed load (drift load) than in larger populations (Kimura et al. 1963). While weakly deleterious mutations can drift all the way to fixation in small populations, these populations have more efficient selection against recessive deleterious variants, as these variants find themselves homozygous more often in small than in large populations. Overall, theory predicts that large populations will harbor more concealed load and less expressed load than smaller populations (Bataillon and Kirkpatrick 2000; Robinson et al. 2023). At the same time, population structure, particularly matings among close relatives, can also reduce concealed load. Inbreeding purges recessive deleterious variation by increasing the selectable phenotypic variance, both by genetic drift and by systematic effects on genotype frequencies (Glemin 2003).

Efforts to study broad patterns of relationship among expressed and concealed load, population history, and genetic diversity have been hampered by the difficulties in measuring the relevant quantities in populations that differ in these respects but are otherwise similar in their genetics and life history. Here, we focus on a set of related species that are extremely similar in basic biological parameters. *Caenorhabditis* nematodes exhibit exceptional morphological and molecular conservatism, right down to cell lineage (Zhao et al. 2008; Memar et al. 2019), developmental gene expression (Levin et al. 2012), gene function (Verster et al. 2014), and chromosome organization and recombination landscape (Stein et al. 2003; Ross et al. 2011; Teterina et al. 2020, 2023; Noble et al. 2021; Sun et al. 2022), and they are well matched in factors such as offspring number and body size.

The best studied *Caenorhabditis* species occur in nature primarily as highly inbred self-fertile hermaphrodites, incapable of mating with one another. In this androdioecious mating system, rare males provide opportunities for outcrossing. The majority of *Caenorhabditis* species, conversely, are gonochoristic, having separate males and females, and are therefore obligate outcrossers. The contrast in mating systems is associated with profound differences in the magnitude of inbreeding depression, which is effectively absent in the selfing species and extreme in the best-studied outcrossers, and genetic diversity, which is depauperate in selfers and preposterous in outcrossers (Dolgin et al. 2007; Dey et al. 2013; Cutter et al. 2019; Braendle and Paaby 2024). Efforts to study gonochoristic *Caenorhabditis* have been severely hampered by the difficulty of making inbred lines in the lab; lines propagated by full-sib mating rapidly suffer extinction (Dolgin et al. 2007; Barriere et al. 2009; Fierst et al. 2015; Teterina et al. 2020).

As inbreeding depression can act as a barrier to mating-system evolution (Lloyd 1979), the levels of segregating recessive deleterious variation in extant male/female *Caenorhabditis* species may be an effective barrier to transitions from obligate outcrossing to selfing. The clade has given rise to extant species with androdioecious mating systems 3 times independently (Kiontke et al. 2011), and the genetic requirements for such mating system transitions are minimal: *C. remanei* can be converted from gonochorist to selfer by reducing the activity of just 2 genes (Baldi et al. 2009).

The phenotypic manifestations of inbreeding depression depend on the nature of the underlying recessive genetic variants, and these in turn are influenced by the interaction of population biology, developmental biology, and selection. Genes acting in males and females experience each of these factors differently, potentially generating sex-biased inbreeding depression (reviewed in Ebel and Phillips 2016). Maternal genetic effects may play a special role: these are sex-limited in their expression and so are expected to harbor twice as much variation at mutation-selection equilibrium as zygotic-effect genes (Wade 1998). They also superimpose among-family selection on top of selection acting among individuals, and they induce positive frequency-dependence when they genetically interact with offspring genotypes, which is compounded by the elevated parent-offspring genetic correlations under inbreeding (Wolf and Wade 2016).

We experimentally estimated loads of concealed and exposed deleterious variation in isolates of several gonochoristic *Caenorhabditis* species. We set out to address 4 questions. First, to what extent do these species vary in their loads of concealed and expressed genetic load? Second, are there species in which the concealed load is sufficiently low that they can be inbred to establish a convenient experimental model species for gonochoristic *Caenorhabditis*? Third, is the concealed load in gonochoristic species large enough to act as an obstacle to evolutionary transitions to selfing? And fourth, does inbreeding depression manifest differently across life stages and sexes?

## Methods

### Establishment of experimental populations

We experimentally inbred 11 isolates of gonochoristic *Caenorhabditis* nematodes and measured the change in cross success with increasing levels of inbreeding. Two isolates of *C. remanei,* EM464 from Brooklyn, New York, and PB219 from Dayton, Ohio, are widely studied exemplars of this cosmopolitan, genetically diverse species and are included as positive controls for the presence of inbreeding load (Dolgin et al. 2007; Barriere et al. 2009; Fierst et al. 2015; Ebel and Phillips 2016; Adams et al. 2022). At the other end of the spectrum, we examined CB4108, a *fog-2(q71)* mutant strain of *C. elegans* (Schedl and Kimble 1988). This completely inbred derivative of the androdioecious lab strain, N2, from Bristol, England, is defective in hermaphrodite self-sperm production and therefore behaves as a gonochorist. This strain is expected to exhibit no inbreeding depression and is included as a negative control. EM464, PB219, and CB4108 were acquired from the *Caenorhabditis* Genetics Center. The remaining 8 experimental populations are 2 strains from each of 4 gonochoristic species from across the Elegans supergroup: *C. panamensis* and *C. becei* from Barro Colorado Island, Panama (Sloat et al. 2022), *C. kamaaina* from Kaua’i, Hawaii (Félix et al. 2014), and *C. remanei* from Okinawa, Japan. We established each population from wild-collected animals via isofemale bottleneck, which isogenizes mitochondrial genotype in each population, followed by expansion to large population sizes (∼10^5^) over several generations prior to cryopreservation of these founder stocks.

### Experiment 1: Estimating load parameters by serial sib mating

We thawed each strain and established large populations on 10 cm NGM-agarose plates seeded with OP50-1 *E. coli* bacteria as a food source. Each population was cleaned of any contaminants by bleaching, preserving population size. Worms went through approximately 8 overlapping generations under these conditions prior to the start of inbreeding. We then initiated 108 independent lines of each wild strain for inbreeding by serial sib-mating and 120 lines for *C. elegans fog-2* mutants (1200 experiments in total). Crosses involved pairing a single L4 (juvenile) male and a single L4 female on a 6 cm NGM-agarose plate seeded with 50 µl of OP50-1. All crosses were performed at room temperature and were carried out by 6 people, each starting simultaneously with 200 anonymized lines in a balanced randomized block design. Each cross yielded L4s of each sex to establish a new cross, or it failed to do so and was scored a line extinction. The primary phenotype scored is thus a binary outcome: does a pair of worms, conditional on having reached the L4 stage, succeed or fail in producing at least 1 L4 offspring of each sex. In addition to this basic cross success phenotype, we analyzed 3 binary (yes/no) subphenotypes: *Copulation*, whether a pair of worms successfully copulates, as demonstrated by the presence of a copulatory plug on the female; *Fertility*, whether a copulation results in laying of embryos; and *Development*, whether any of the embryos develop to the L4 stage. Inbreeding was stopped in the 21st generation. Surviving lines were cryopreserved and are available for further analysis.

Morton et al. (1956) modeled the probability of an individual's survival under inbreeding as a function of several factors, including the effects of deleterious variants segregating at Hardy-Weinberg Equilibrium, the effects of environmental factors, and the effects of recessive deleterious variants homozygosed by inbreeding. Assuming that the effects of each locus and environmental factor are small and independent, *S*, the probability of survival, takes on a simple form, *S* = *e^–A – BF^*. *F* is the inbreeding coefficient, the probability that alleles in an individual are identical by descent. In a regression of −*ln(S)* on *F*, the intercept *A* represents the effects of environmental factors, including those that depend on fixed load, and the effects of segregating load in the absence of inbreeding. *B* represents the effects of inbreeding.

We modified this approach to model *R*, the probability of a successful reproduction event, rather than *S*. Because reproduction requires that each of 2 worms succeeds, the coefficients *A_R_* and *B_R_* are approximately twice the magnitude of Morton et al.'s *A* and *B* for survival probability, as shown in Supplementary File 1.

To fully leverage the binary nature of the data—each cross succeeded or failed—we performed logistic regression in R (R Core Team 2024) to estimate the relationship between inbreeding and each phenotype (Supplementary File 2). Assuming successes are binomially distributed with success probability *R*, we model the log of the odds ratio *ln* $(R/(1-R))=\;\beta_{0}+\beta_{1}F$, implemented using a generalized linear model with a logit link function. The coefficients can be converted into estimates of *A_R_* and *B_R_* as *A_R_* =− *ln*(*R_F_*_=__0_) = −*ln*($e^{\beta_{0}}/(1+e^{\beta_{0}})$), and *B_R_* = −*ln*(*R_F_*_=__1_/*R_F_*_=__0_) = −*ln* $\left(\left(e^{\beta_{0}+\beta_{1}}/(1+e^{\beta_{0}+\beta_{1}}))/(e^{\beta_{0}}/(1+e^{\beta_{0}})\right)\right)$. For each strain, we tested the significance of the $\beta_{1}$ estimate using a *z*-test implemented using the *summary(glm())* functions in R.

Defining the starting population as our reference, the first generation of each cross is between outbred (*F* = 0) individuals, yielding outbred embryos (*F* = 0). Crosses in the next generation therefore also represent *F* = 0 crosses. Their offspring are inbred (*F* = 0.25), and sequential generations have inbreeding coefficients that increment each generation according to the recursion *F_t_* = 0.25 * (1 + 2*F_t_*_-1_ + *F_t_*_-2_) (Lynch and Walsh (1998), eq. 10.5b).

### Experiment 2: Relative fitness of outbred populations and inbred lines

We estimated fitness from reproductive schedules for 6 females from each of nineteen strains: 5 outbred founders and 2 to 3 inbred lines derived from each (Supplementary File 3). The inbred lines were selected at random from the collection of inbred lines from experiment 1.

We thawed each strain and maintained them on food at large population sizes for several generations prior to the start of the experiment. We isolated L4 animals of each sex and left them to mature. We then paired an adult male and an adult female for 5 hours, after which we removed the male. The female was left to lay embryos until 6 h from the initial pairing and was subsequently transferred to a new plate every 8 h until it had stopped laying embryos. After 60 to 80 h of development, we counted the adult progeny on each plate (ie dead embryos and arrested larvae were not counted). The experiment was performed at room temperature in a single block by a single investigator, blinded to strain identity, and the order of the 120 females was randomized.

Seven females were censored from analysis because they failed to mate or they crawled onto the edge of the plate and desiccated before the conclusion of egg laying. For the remaining 113 females, we counted the total number of progeny that developed to adulthood and, following Dolgin et al. (2007), we estimated relative fitness using the Lotka-Euler equation for age-structured populations (Charlesworth 2009, eq 1.32). This approach estimates the intrinsic rate of growth for a population with overlapping generations, under conditions of age-structure equilibrium. The population growth parameter *r* was chosen to set the mean fitness of the outbred founder females to 1, separately for each founder (ie comparisons are between founders and their descendant inbred lines, not among founders or unrelated inbred lines). Note that fitness measured in this way incorporates the reproductive schedule starting from adulthood; development time to reproductive maturity for the assayed females was not measured but likely contributes to fitness. Relative fitness is correlated with number of offspring (Pearson's correlation coefficient = 0.87) but better captures the proliferative ability of each animal in the case of overlapping generations.

### Experiment 3: Tests for complementation and maternal effects

We used scanner-based population growth-rate assays (Tintori et al. 2024) to estimate fitness complementation and maternal effects for 2 inbred lines of *C. panamensis,* QG1819 and QG1892, both derived from founder isofemale line QG702.

The strains were thawed and raised at 25 °C on NGMA plates seeded with OP50-1. Populations were then bleached and the resulting L4s picked to establish 4 types of cross: QG1819xQG1819, QG1892xQG1892, QG1819xQG1892, and QG1892xQG1819. When the progeny of these crosses had developed to L4, they were separated by sex and allowed to mature to adulthood overnight. The resulting first-day adults were then picked to 3.5 cm assay plates containing NGMA with 10 µg/ml nystatin and 50 µg/ml streptomycin. Assay plates were seeded with 200 µl of OP50-1, concentrated 10 × from an overnight culture. Six types of plate were set up: self crosses of QG1819 and QG1892, reciprocal crosses between QG1819 and QG1892, and self crosses of each class of F_1_, those with QG1819 mothers and those with QG1892 mothers. Each assay plate received 2 males and 2 females, with 10 replicate plates per cross type.

Plates were randomized across acrylic plate-holders in 4 manually refocused Epson V800 and V850 scanners, all inside a 25 °C incubator. Scanners recorded 1200-dpi images once per hour for 8 d. Images were processed and statistics derived following the *popscan* pipeline (Tintori et al. 2025). We compared log(hours to resource exhaustion) among strains using Wilcoxon tests.

## Results

**Fig. 1.**
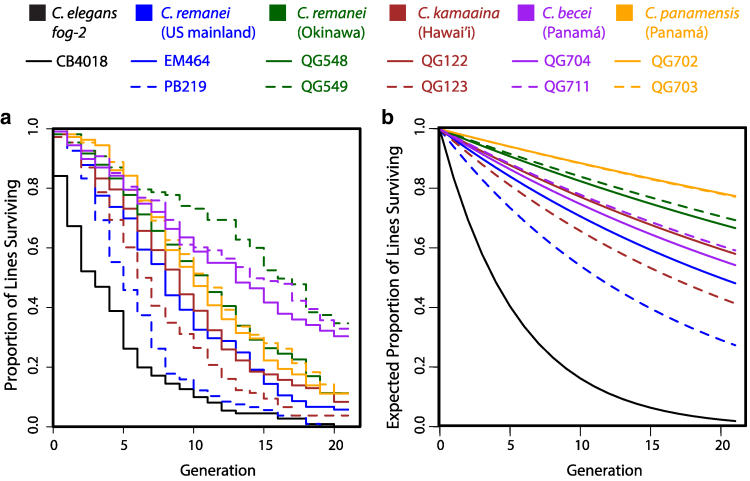
a) During the course of 20 generations of serial full-sib mating, the majority of lines for each of 11 *Caenorhabditis* isolates went extinct. The 2 lines for each color represent the 2 different founding isolates tested for each wild population. b) In the absence of inbreeding depression, the expected survival curves follow exponential decay, reflecting a constant probability of cross failure. The difference between panels 1A and 1B demonstrates the extent of concealed genetic load, which is significant for every strain except the control strain, *C. elegans fog-2*. This strain began the experiment fully inbred, and its steep die-off reflects its high but constant rate of cross failure. See Table 1 for estimates, errors, and *P*-values.

We set up crosses between single male and female worms and scored whether the worms mated, whether the female laid embryos, and whether those embryos hatched and developed to the L4 stage. If the cross produced L4 progeny, 1 pair of male and female worms were randomly selected for the next generation; if the cross failed, we recorded the step at which it failed: copulation, fertility, or development (Supplementary Table 1). Of 1200 such experiments, 157 (13%) successfully reproduced through 20 consecutive generations of full-sib matings; the remaining lines suffered extinction during the experiment, as shown in Fig. 1a. The distribution of extinctions across the resulting 11,892 crosses revealed the patterns of expressed and concealed genetic load in 11 strains of gonochoristic *Caenorhabditis* nematode.

We used logistic regression of the outcome of each cross on the inbreeding coefficient *F* to estimate the load for each isolate (Table 1). Our analysis yields estimates of logistic regression coefficients, $\beta_{0}$ and $\beta_{1}$, which are related to the population-genetic quantities *A_R_* and *B_R_* defined by Morton et al. (1956). *A_R_* includes the effects of expressed load, the reduction in the log of fitness relative to a mutation-free individual, in the absence of inbreeding depression, and *B_R_* represents the concealed load, the slope of the decline in log fitness as a function of inbreeding.

**Table 1.** Estimates of expressed and concealed load.

Species	Strain	Locality	$\beta_{0}$	SE($\beta_{0})$	$\beta_{1}$	SE($\beta_{1})$	*A_R_*	*B_R_*	*P* ($\beta_{1}$ = 0)
*C. elegans*	*fog-2*	NA	1.517	0.156	−0.219	0.313	0.198	0.043	0.484
*C. remanei*	EM464	New York	3.330	0.272	−1.816	0.376	0.035	0.164	<10^−5^
*C. remanei*	PB219	Ohio	2.730	0.220	−1.974	0.356	0.063	0.322	<10^−7^
*C. remanei*	QG548	Okinawa	3.935	0.334	−2.123	0.427	0.019	0.132	<10^−6^
*C. remanei*	QG549	Okinawa	4.038	0.372	−1.320	0.464	0.017	0.046	0.004
*C. kamaaina*	QG122	Kaua’i	3.633	0.299	−2.048	0.398	0.026	0.160	<10^−6^
*C. kamaaina*	QG123	Kaua’i	3.134	0.252	−1.931	0.368	0.043	0.220	<10^−6^
*C. panamensis*	QG702	Panamá	4.403	0.380	−2.798	0.478	0.012	0.171	<10^−8^
*C. panamensis*	QG703	Panamá	4.391	0.383	−2.675	0.478	0.012	0.153	<10^−7^
*C. becei*	QG704	Panamá	3.513	0.307	−0.899	0.402	0.029	0.041	0.025
*C. becei*	QG711	Panamá	3.671	0.325	−0.985	0.419	0.025	0.041	0.019

For each strain, the table shows the logistic regression parameter estimates and their standard errors, as well as the associated point estimates of the parameters *A_R_* and *B_R_* from Morton et al. (1956). The final column reports the *P* value for a test of the hypothesis that $\beta_{1}$ = 0; significant results point to the presence of concealed genetic load.

The estimates of *A_R_* allow us to plot the expected survival curves in the case where cross success rate is constant (Fig. 1b). The differences between the curves in Fig. 1, a and b reveal the excess cross failures caused by inbreeding.

Every wild isolate showed significant concealed load, manifesting as decrease in reproductive success with increasing inbreeding (*i.e*. $\beta_{1}$ < 0). The negative control strain, *C. elegans fog-2*, which was completely inbred from the start, showed the expected absence of significant inbreeding depression. The severity of inbreeding depression varied considerably among species, and, in some cases, among isolates within species (Table 1).

Several isolates showed considerable expressed load in the absence of inbreeding (i.e. *A_R_* > 0). These values, which reflect the fixed and segregating load carried by each isolate, were small in magnitude (Table 1). The exception is the *C. elegans fog-2* control, for which *A_R_* was approximately 0.2; this value corresponds to a cross failure rate of about 18%. This result was anticipated, as *C. elegans* mates rarely in nature and its mating ability is diminished relative to obligately outcrossing species.

Crosses failed at 3 different steps in the reproductive process: copulation, fertility, and development. Analyzing these steps 1 by 1, we found that the expressed load was largely attributable to the copulation step; conditional on successful copulation, we saw negligible expressed load for fertility and growth (Supplementary Table 2). In contrast, most strains showed significant inbreeding load for each step of the process. That is, we observed inbreeding depression for copulation success, and conditional on copulation success we observed inbreeding depression for fertility, and conditional on fertility we observed inbreeding depression for embryonic and larval growth.

Isolates that show low inbreeding depression by our binary reproductive success metric may nevertheless suffer considerable quantitative reductions in fitness. To assay fitness quantitatively, we selected 5 outbred founder strains (EM464, QG549, QG123, QG702, and QG704) and 2 to 3 random inbred lines derived from each. For these 19 strains, we estimated the fitness of the inbred derivatives relative to their outbred ancestors by measuring the reproductive schedules and number of offspring that developed to adulthood (Supplementary Table 3).

**Fig. 2.**
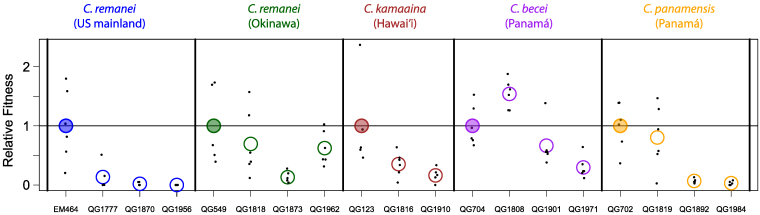
Inbred lines have reduced and heterogeneous fitness relative to their ancestor. The *y* axis shows the relative fitness of the 5 ancestors and several of their inbred descendants. Each ancestor's mean relative fitness (filled circles) is set to 1. Each point is a single mated female, and the circles represent strain means.

In most cases, inbred lines exhibited significantly reduced fitness relative to their outbred ancestor (Fig. 2). For *C. remanei* EM464, the fittest of the 3 descendant inbred lines had a relative fitness of only 0.135. Each of the other founders produced a mix of severely damaged (relative fitness <0.5) and reasonably fit inbred descendants, and in 1 case, a line derived from *C. becei* QG704, inbred descendants had higher average relative fitness than their outbred ancestors. For 3 of 5 founders, the inbred lines from each exhibited significant heterogeneity in fitness (Kruskal-Wallis tests for effect of strain, *P* < 0.05).

**Fig. 3.**
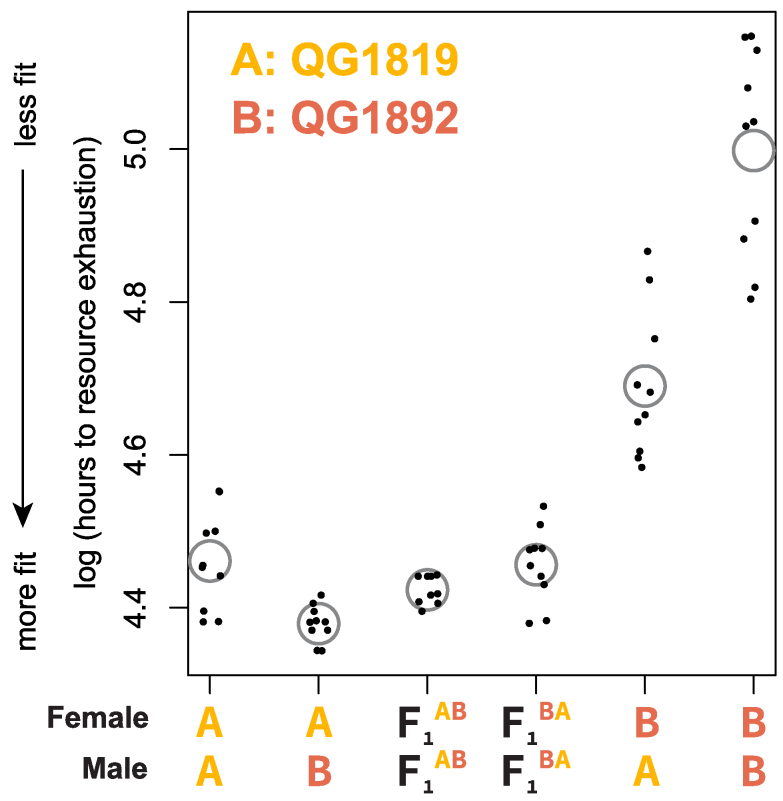
Crosses between related inbred lines of *C. panamensis* show complementation and maternal effects. The *y* axis shows the log of the number of hours required for a population founded by 2 males and 2 females to exhaust a fixed quantity of resources; the population at that point typically includes thousands of grandchildren of the founders. Points lower on the *y*-axis represent populations with faster population growth and consequently greater fitness. Each point is a population and gray circles indicate means. The F_1_s are the product of matings between females of strain A and males of strain B (F_1_^AB^) or the reciprocal cross (F_1_^BA^).

If inbred lines derived from a single founder population have fixed different haplotypes, crosses between them should result in complementation of recessive alleles and a restoration of fitness. We tested for complementation using 2 *C. panamensis* lines derived from founder QG702. The 2 inbred lines, QG1819 and QG1892, differ dramatically in relative fitness in our reproductive schedule assay (Fig. 2), and so are likely to have fixed different haplotypes. We used flatbed scanner assays to estimate population fitness in these 2 lines, reciprocal crosses between them, and crosses between F_1_ animals from each of the reciprocal crosses (Fig. 3; Supplementary Table 4). These assays recapitulated the large difference in fitness between the inbred lines that we observed in Fig. 2 (Wilcoxon test, *P* = 1.1 × 10^−5^), and crosses result in a dramatic increase in fitness relative to the less fit parent, consistent with complementation.

Notably, reciprocal crosses between the inbred lines differ substantially (*P* 1.8 × 10^−4^), consistent with sex-specific inbreeding depression: fitness is much higher when the high-fitness inbred line is the female in the cross. This directional effect is reduced or absent when crosses start with the F_1_ worms from the reciprocal crosses (*P* = 0.10). Given our experimental design, this pattern implicates female-biased inbreeding depression that results in some mixture of reduced brood size or ovulation rate (features of the maternal phenotype) or reduced viability or developmental rate in the F_1_s (maternal-effect features of the pre-adult F_1_ phenotype). The reciprocal crosses also differ in the genotypes of the X chromosomes carried by the male progeny, which may contribute to phenotypic difference.

## Discussion

We found that all tested gonochoristic *Caenorhabditis* isolates carry a significant burden of recessive deleterious alleles, with fitness declining as inbreeding increases. These results are congruent with findings from experimental analyses of *C. remanei* and *C. brenneri* (Dolgin et al. 2007; Barriere et al. 2009; Fierst et al. 2015; Ebel and Phillips 2016; Adams et al. 2022), and with the commonplace experience of every researcher working with gonochoristic *Caenorhabditis* strains.

The severity of the experimentally exposed inbreeding depression varies substantially among species and among isolates within species. In *C. remanei*, a widely distributed generalist species, we observe higher concealed load in 2 strains from North America than in 2 strains from Okinawa. This observation is consistent with the theoretical expectation that reduced population size, associated here with life on a small island, should facilitate purging of concealed load (Dussex et al. 2023). However, *C. kamaaina* isolates, from an island of similar size but greater remoteness (Kauai), has a very high concealed load. *C. kamaaina* is likely an ancient Hawaiian endemic, while Okinawan *C. remanei* may be a recent peripheral isolate; thus population age may be playing a key role in the architecture of recessive load. *C. becei* and *C. panamensis*, nearly indistinguishable species that live in sympatry in Panamá (Sloat et al. 2022; their full geographic ranges are unknown), differ in their estimated loads by more than a factor of 3. These disparate levels of concealed load reinforce the view that *Caenorhabditis* nematodes are a useful model for discovering the population-biological factors that shape recessive deleterious variation.

A key practical finding is that *C. becei* exhibits a manageably low concealed load, with a large number of its experimental lines (70/216) surviving 20 generations of sib mating. By comparison, only 10/216 lines of the North American *C. remanei*, the classic model gonochoristic *Caenorhabditis*, survived the process. In addition, some of the *C. becei* inbred lines retained high fitness. Given these observations, we are now developing tools for experimental studies of *C. becei,* to establish it as a tractable model for obligately sexual *Caenorhabditis* species and for genetic analysis of recessive deleterious alleles (Salome-Correa et al. 2025).

Some isolates also have signatures of expressed genetic load prior to systematic inbreeding, perhaps as a symptom of their original derivation by isofemale bottleneck. By far the greatest level of expressed load is found in *C. elegans*. As expected, the *fog-2* mutant strain exhibits no significant inbreeding depression, because it is already fully homozygous. But it is profoundly deficient at mating, failing in 18% of pairings. Some fraction of this deficit may reflect the fixation of deleterious alleles in the ancestry of *C. elegans*, associated with the evolution of selfing or with the accumulation of deleterious alleles during long periods of evolution with little outcrossing and reduced effective population sizes (Cutter 2019). However, the step in our assay at which *C. elegans* fails—copulation—suggests that the expressed load in *C. elegans* may simply reflect relaxed selection on male function and mating ability in this androdioecious species, which almost always reproduces by self-fertilization in nature (Palopoli et al. 2008; Noble et al. 2015; Yin et al. 2018; Cutter et al. 2019).

In theory, the load of recessive deleterious alleles carried by a gonochoristic population presents an obstacle to mating-system evolution via invasion by selfing alleles (Lloyd 1979; Charlesworth and Charlesworth 1987). Although we observed significant decreases in the probability of successful reproduction in each of the 10 gonochoristic isolates we examined, the magnitudes of these decreases were not large. Estimated cross success at *F* = 1 ranges from 69% in PB219 to 94% in QG549. Our experiments involved single-worm-pair matings, but in natural populations, typical brood sizes in the high hundreds would buffer the extinction risk of even dramatically lowered per-pair reproductive success probabilities. The worm-breeder's frustration with experimental inbreeding may largely reflect our historic reliance on strict sib-mating designs.

If cross success were a comprehensive measure of individual fitness, inbreeding depression in these isolates is sufficiently small that an allele causing selfing should be able to invade. Because the transition to selfing in *Caenorhabditis* is genetically simple (Baldi et al. 2009), these data suggest that the true fitness costs to inbreeding may greatly exceed those measured by our assay sib-mating assay. We observed such costs in our analysis of reproductive schedules for fully inbred individuals (Fig. 2). In many cases—in all 3 genotypes examined for the North American *C. remanei*—relative fitness was substantially lower than the canonical value required to prevent invasion of selfing (Lloyd 1979). In most species, however, we observed substantial among-line variation in the reduction in fitness, with some lines performing very well. This variance among individuals in the burden of recessive deleterious alleles creates the potential for facile transition to mixed-mating and ultimately selfing (Lande and Schemske 1985; Ebel and Phillips 2016; Brown and Kelly 2020). Such transitions would potentially be aided by progressive purging of the recessive load and benefits to selfers from mating assurance in patchy environments. Inbreeding depression may not be the whole explanation for the rarity of selfing species in *Caenorhabditis*.

Our experiments allow us to localize inbreeding depression in several different places in the life cycle. We found that, for most isolates, inbreeding load was detectable at the steps of mating, fertility, and embryonic and larval growth. In the 1 pair of *C. panamensis* inbred lines we examined in reciprocal crosses, we detected a strong female bias in the magnitude of inbreeding depression. These findings generalize the results of previous studies in *C. remanei* (Dolgin et al. 2007; Ebel and Phillips 2016).

Female-specific effects in our assay likely represent a mixture of direct and maternal-effect phenotypes. Anecdotally, each of the 10 wild isolates we inbred produced at least 1 line that went extinct when the female laid a large number of embryos, all of which failed to hatch. This is a typical maternal-effect pattern, with no segregation within the brood. Maternal-effect genetic variation is abundant within and among *Caenorhabditis* species (Farhadifar et al. 2015, 2020; Ben-David et al. 2017; Noble et al. 2021), as is variation in zygotic sensitivity to maternal genetic perturbations (Paaby et al. 2015; Torres Cleuren et al. 2019). While maternal-effect inbreeding depression in vertebrates putatively operates through changes in maternal care (*e.g.* Sin et al. 2021), the cytoplasmic basis of maternal genetic effects in *Caenorhabditis* provides a promising path toward understanding recessive genetic variation affecting early development.

## Supplementary Material

jkaf200_Supplementary_Data

## Data Availability

All worm strains are available from the authors. All data are in the Supplementary tables. Supplemental material available at G3 online.
