# Supplementary material for: Variation in inbreeding depression within and among Caenorhabditis species
Source: G3 (Bethesda). 2025 Aug 29;15(12):jkaf200. doi: 10.1093/g3journal/jkaf200 (PMC12836119; doi:10.1093/g3journal/jkaf200)
Supplement: jkaf200_Supplementary_Data [file jkaf200_Supplementary_Data.zip › Supplementary_Material_Legends_G3-2025-406147.docx]

**SUPPLEMENTARY FILES**

**Supplementary Table 1.** Raw data reporting the results of the 1200 sibmating experiments described in the manuscript. For each experiment, the table reports the experiment number (used to blind the experimenters to the strain identity), an identifier for the worm picker who carried out the experiment, and the worm strain and species. The result for each experiment is recorded as the last generation of the experiment in which the strain was observed alive. The status column indicates whether the strain died in that last recorded generation (status = 1) or whether it was still alive (status = 0). The columns Copulation, LaidEmbryos, and Young Adults record for each experiment whether in the final generation the line successfully copulated, laid embryos, and produced offspring that developed to the Young Adult stage. The data in this file underlie Figure 1, Table 1, and Supplementary Table 2.

**Supplementary Table 2.** For each trait (Reproduction, Copulation, Fertility, Development), the table reports the estimate and standard error of $\beta_{0}$ and $\beta_{1}$, the estimates of *A_R_* and *B_R_*, and the p-value for $\beta_{1}$ for each of the 11 isolates.

**Supplementary Table 3.** For each of 120 individual females tracked through their reproductive lives, the table reports the strain, species, experimental plate, and number of male and female adults that developed from each of eight 8-hour time blocks. For example, columns 1F and 1M record the Female and Male progeny that developed to adulthood from embryos laid during the 1^st^ time block. These data underlie Figure 2.

**Supplementary Table 4.** For each of 59 experimental plates tracked by flatbed scanner for population growth, the table records the genotype (*treatment*), the phenotype (*sd_hours_to_starve*, hours to resource exhaustion, measured as the peak of the standard deviation of pixel intensity), the replicate plate for the treatment (*rep*, 1-10), the scanner used to score the plate (*scanner*, 5-8), and the position of the replicate plate on the scanner (*pos*, 1-9). These data underlie Figure 3.

**Supplementary File 1. Comparison of Reproduction Probability to Morton *et al.*’s Survival Probability.**

**Supplementary File 2.** This file contains annotated R code that performs the reported analyses of the sib mating experiments and reproduces Figure 1 and Table 1 and Supplementary Table 2, starting with the raw data from Supplementary Table 1.

**Supplementary File 3.** This file contains annotated R code that performs the reported analyses of relative fitness and reproduces Figure 2, starting with the raw data from Supplementary Table 3.
